# Examining Sleep Habits and Associated Lifestyle Factors in Adolescents: A Population‐Based Study

**DOI:** 10.1002/brb3.70885

**Published:** 2025-10-09

**Authors:** Claire Chenwen Zhong, Mingtao Chen, Zhaojun Li, Zehuan Yang, Vera M. W. Keung, Amelia Lo, Calvin Cheung, Junjie Huang, Martin C. S. Wong

**Affiliations:** ^1^ JC School of Public Health and Primary Care, Faculty of Medicine The Chinese University of Hong Kong Hong Kong SAR China; ^2^ Centre for Health Education and Health Promotion, Faculty of Medicine The Chinese University of Hong Kong Hong Kong SAR China

**Keywords:** adolescent, associations, health behaviors, sleep

## Abstract

**Introduction:**

This study aimed to examine the associations between adolescent characteristics and sleep habits in Hong Kong.

**Methods:**

A cross‐sectional study was conducted among secondary school students in Hong Kong. Data on sociodemographics, health behaviors, mental toughness, and sleep patterns were collected. Univariable and multivariable logistic regression analyses were used to identify factors associated with late bedtime, insufficient sleep duration, and late wake‐up time.

**Results:**

Among 1345 adolescents surveyed, 60.1% reported late bedtime, 56.6% experienced insufficient sleep duration (<8 h), and 34.1% reported late wake‐up time. In multivariable models, older age was consistently associated with adverse sleep habits (aOR_late wake‐up_ = 1.64, 95% CI: 1.23–2.18, *p* = 0.001; aOR_late bedtime_ = 2.69, 95% CI: 1.98–3.64, *p* < 0.001; aOR_insufficient duration_ = 1.74, 95% CI: 1.31–2.31, *p* < 0.001). Exceeding recommended screen time was linked to later bedtimes (aOR_video_ = 1.46, 95% CI: 1.14–1.88, *p* = 0.003; aOR_game_ = 1.42, 95% CI: 1.10–1.84, *p* = 0.007; aOR_social media_ = 1.78, 95% CI: 1.38–2.29, *p* < 0.001) and insufficient sleep duration (aOR_social media_ = 1.87, 95% CI: 1.46–2.39, *p* < 0.001). Daily breakfast consumption was consistently associated with lower odds of late bedtime (aOR = 0.49, 95% CI: 0.39–0.63, *p* < 0.001), insufficient sleep duration (aOR = 0.56, 95% CI: 0.44–0.71, *p* < 0.001), and late wake‐up time (aOR = 0.69, 95% CI: 0.54–0.88, *p* = 0.002). Alcohol consumption was associated with higher odds of insufficient sleep duration (aOR = 1.80, 95% CI: 1.10–2.96, *p* = 0.020) and lower odds of late wake‐up (aOR = 0.60, 95% CI: 0.37–0.96, *p* = 0.032). Male students had lower odds of insufficient sleep (aOR = 0.69, 95% CI: 0.54–0.87, *p* = 0.002) but higher odds of waking up late (aOR = 1.75, 95% CI: 1.37–2.24, *p* < 0.001).

**Conclusion:**

This study identified high rates of adverse sleep habits among Hong Kong adolescents and demonstrated strong associations with age, screen use, dietary behavior, and other lifestyle factors. These findings highlight the need for targeted interventions—such as digital use guidelines and school‐based breakfast programs—to promote healthy sleep patterns during adolescence.

## Graphical abstract

This study identified high rates of adverse sleep habits among Hong Kong adolescents, with 60.1% of adolescents reporting late bedtime, 56.6% having insufficient sleep duration, and 34.1% reporting waking up late. The multivariable analysis indicated that older age and excessive screen use are key risk factors, while breakfast consumption works as a protective factor. These findings underscore the need for targeted interventions—such as digital hygiene promotion and school‐based sleep education—to foster healthier sleep routines among adolescents.

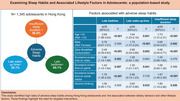

## 1 Introduction

Adverse sleep habits generally refer to a series of behaviors that may have a negative impact on sleep quality, including inadequate sleep duration, sleeping at an inappropriate time, and sleep disorders, which are common types of adverse sleep habits (Centers for Disease Control and Prevention 2025). In recent years, an increasing number of studies have highlighted the adverse physical or mental health outcomes associated with adverse sleep habits in the adolescent population. For instance, a study from Korea involving 6048 adolescents found that adolescents who were sleep deprived had a higher risk of cardiovascular disease and were more likely to be obese or overweight (Seo and Shim 2019). Additionally, substantial evidence indicates a strong association between poor sleep habits in adolescents and emotional issues, along with risk‐taking behaviors. A previous study involving 10,123 adolescents aged 13–18 in the United States revealed that delayed bedtimes and insufficient sleep duration are linked with the odds of symptoms of anxiety, depression, and suicidal ideation (Zhang et al. 2017). Similarly, a systematic review and meta‐analysis indicated that insufficient sleep contributes to risk‐taking behaviors such as smoking, violent behavior, and substance use (Short and Weber 2018).

In light of the potential adverse consequences of poor sleep habits, authorities have issued recommendations on sleep schedules tailored to individuals at various life stages. The American Academy of Sleep Medicine recommends that adolescents require approximately 8–10 h of sleep per day to meet the physiological demands of adolescence (Paruthi et al. 2016). However, adherence to this recommendation among the adolescent population has not been favorable globally. As reported by the US Centers for Disease Control and Prevention, 77% of adolescents in the United States did not meet the recommended sleep duration of 8 h in 2021, with the prevalence being even more pronounced among females (Centers for Disease Control and Prevention 2024). A study from Hong Kong indicated a similar phenomenon, with about 61% of adolescents aged 11–18 reporting less than 8 h of sleep per day (Huang et al. 2023).

The causes of adolescents’ poor sleep habits are complex, involving both physiological and social factors. On one hand, the delayed secretion of melatonin during adolescence results in later sleep onset (Carskadon et al. 2004). On the other hand, the conflict between adolescents’ daytime roles and their personal needs is also significant. A qualitative study from Slovakia suggests that one of the most important reasons for adolescents’ delayed sleep is that the daytime role of being a student restricts their use of personal time, and as a result, they perceive nighttime as a compensatory opportunity (Watling et al. 2017).

Significant efforts have been made to explore potentially feasible interventions to improve the sleep habits of adolescents, including health education on sleep habits and delayed school start times (Chung et al. 2017; Borisenkov et al. 2022). Essentially, these interventions are designed to increase the amount of time adolescents spend sleeping; however, research has found that their effectiveness is inconsistent across different sleep habit patterns (Chung et al. 2017; Vedaa et al. 2012). Given the potential relationship between various types of sleep habits and personal characteristics in adolescents (Moore et al. 2011), highlighting the importance of adolescent traits in sleep habits is crucial for improving and customizing interventions in the future.

However, current studies have primarily focused on the health outcomes of adverse sleep habits (Lunsford‐Avery et al. 2022), trends in sleep patterns (Chen et al. 2024), or the role of adolescent characteristics in Western cultures (Moore et al. 2011). The role of individual characteristics in adolescents has not yet been adequately examined in the Asian context. Therefore, this study aims to thoroughly assess the association between various factors and different sleep habits among adolescents in Hong Kong. This will help identify target groups for intervention and facilitate the development of targeted interventions.

## 2 Methods

### 2.1 Study Design and Participants

This cross‐sectional study investigated factors associated with sleep patterns among secondary school students. Participants were recruited from Form 1 to Form 4 in local secondary schools that participated in the “Health Education Built‐on Project” organized by the Centre for Health Education and Health Promotion at the Chinese University of Hong Kong. The study design and reporting adhered to the STROBE (Strengthening the Reporting of Observational Studies in Epidemiology) guidelines (Von Elm et al. 2007).

### 2.2 Data Collection

An invitation letter outlining the study objectives was distributed to 20 participating schools. Upon receiving institutional approval and written informed consent from both students and their parents, printed questionnaires were administered during class under standardized instructions provided by trained teachers. Participation was voluntary and anonymous. Completed surveys were sealed and returned directly to the research team to ensure confidentiality. Ethical approval was obtained from the Survey and Behavioural Research Ethics Committee of the Chinese University of Hong Kong (SBRE‐24‐0029).

### 2.3 Survey Instrument

A self‐administered questionnaire was developed to collect information on demographic and socioeconomic background, sleep‐related behaviors, health practices, psychological attributes, and health literacy. Details of the questionnaire are provided in Table S1.

Socioeconomic status (SES) was measured using the Family Affluence Scale II, which is a composite score derived from household possessions and travel frequency to determine the SES of participants (Liu et al. 2012). In accordance with recommendations and to keep consistent with previous studies (Liu et al. 2012; Chatelan et al. 2023; Currie et al. 2008), participants were classified into low (scores 0–3), medium (4–5), or high (6–7) SES, in order to better examine the effect of relative or approximate SES position.

The primary outcomes of interest were sleep behaviors, including self‐reported bedtime, wake‐up time, and total sleep duration over the past month. Bedtime was categorized as “early” if it occurred before 11:00 p.m. and “late” if at or after 11:00 p.m.; wake‐up time was defined as “early” if before 7:00 a.m. and “late” if at or after 7:00 a.m.; sleep duration was dichotomized as “sufficient” (≥8 h per night) and “insufficient” (<8 h), in line with international sleep recommendations for adolescents (Paruthi et al. 2016).

The measurement of key covariates was adapted from the Global School‐based Student Health Survey (GSHS) developed by the WHO (2025), including domains in dietary habits (daily breakfast, adequate fruit and vegetable intake), physical activity, risk behaviors (smoking and alcohol use in the past 30 days), weight perception, and screen exposure across three domains: video content, electronic gaming, and social media use. Of these, for analysis purposes, participants who reported consuming ≥1.5 bowls (i.e., three servings) of vegetables and ≥2 servings of fruit per day were categorized as having adequate vegetable and fruit intake, respectively, according to the food consumption guidelines issued by the Centre for Health Protection in Hong Kong (Centre for Health Protection 2017). The physical activity is defined as ≥60 min of moderate to vigorous activity per day, as recommended by the WHO (2020). Participants also rated their weight perception on a 5‐point scale, and the following response set is available: *Very underweight* (Score 1), *Slightly underweight* (2), *About the right weight* (3), *Slightly overweight* (4), and *Very overweight* (5). For analytical purposes, participants with scores 4–5 were classified as self‐reported obese, which aligns with a previous study (Bhurtun and Jeewon 2013). In addition, participants who reported ≥2 h of screen exposure on video content, electronic gaming, or social media per day on weekdays, on average, were categorized as having excessive screen time exposure, as recommended by existing guidelines (Tremblay et al. 2016).

Mental toughness was assessed using the validated instrument Mental Toughness Scale for Adolescents developed by McGeown and colleagues (2018), with 18 items across six domains, including challenge (refers to seeking opportunities for self‐development), interpersonal confidence (confidence in social situation), confidence in abilities (confidence in facing difficulties), emotion control (ability to regulate emotions), life control (beliefs in shaping one's own life), and commitment (belief in and ability to achieve goals), with a total score ranging from 18 to 72. This scale was adapted based on the 4Cs model of mental toughness, focusing more specifically on assessing the association between adolescents’ individual attributes and behavioral tendencies or psychological outcomes under an educational context. Previous research has confirmed the significance of the 4Cs model of mental toughness in studying adolescent health behaviors, such as sleep and psychological well‐being, indicating consistent association on health outcomes among subdomains and overall mental toughness level (McGeown et al. 2018; Lambert et al. 2022; Brand et al. 2014). For analytical purposes, participants were dichotomized into “low” (≤46) and “high” (≥47) levels of mental toughness based on the median cutoff score to capture differences between participants on different characteristics, allowing for a more precise delineation of potential risk groups (DeCoster et al. 2009).

Health literacy was measured using the Health Literacy Measure for Adolescents (Ghanbari et al. 2016), a 44‐item instrument covering eight dimensions of health‐related knowledge and behavior. Scores were categorized as low (0–66) or high (66.1–100), as recommended by the scale developer.

### 2.4 Statistical Analysis

All statistical analyses were conducted using R version 4.2.1. Descriptive statistics were computed to characterize the sample. Between‐group comparisons (e.g., sufficient vs. insufficient sleep) were performed using Chi‐square tests for categorical variables and independent *t*‐tests for continuous variables. To identify factors associated with sleep outcomes, univariable and multivariable logistic regression analyses were conducted. Variables included in the models were age, sex, SES, smoking, alcohol use, breakfast habits, fruit and vegetable intake, physical activity, each type of screen time, weight perception, mental toughness, and health literacy. Adjusted odds ratios (aORs) with 95% confidence intervals (CIs) were reported. Statistical significance was set at *p* < 0.05. Model adequacy was assessed using the Hosmer–Lemeshow goodness‐of‐fit test.

## 3 Results

### 3.1 Participants Characteristics

Table 1 illustrates the sociodemographic characteristics and descriptive statistics of the participants. A total of 1345 adolescents were included in this study, comprising 757 (56.3%) females and 588 (43.7%) males. The majority of adolescents were under 13 years of age (43.5%, *n* = 585), and nearly half of them came from medium‐SES families (49.2%, *n* = 662). In addition, most adolescents claimed as nonsmokers (98.9%, *n* = 1330) and without alcohol consumption (92.7%, *n* = 1247). More than 80% of adolescents reported insufficient intake of vegetables (85.9%, *n* = 1156) and fruit (83.5%, *n* = 1123), as well as physical inactivity (87.1%, *n* = 1171). Detailed information was shown in Table 1.

**TABLE 1:** Participant characteristics by bedtime.

	Overall	Bedtime = Early	Bedtime = Late	*p*
*n*	1345	536 (39.9%)	809 (60.1%)	
Age group				**< 0.001**
< 13 years	585 (43.5)	285 (48.7)	300 (51.3)	
13–14 years	379 (28.2)	156 (41.2)	223 (58.8)	
>14 years	381 (28.3)	95 (24.9)	286 (75.1)	
Sex				**0.009**
Female	757 (56.3)	278 (36.7)	479 (63.3)	
Male	588 (43.7)	258 (43.9)	330 (56.1)	
SES (0–9)				0.304
Low (0–2)	224 (16.7)	85 (37.9)	139 (62.1)	
Mid (3–5)	662 (49.2)	255 (38.5)	407 (61.5)	
High (6–9)	459 (34.1)	196 (42.7)	263 (57.3)	
Smoking				0.782
No	1330 (98.9)	529 (39.8)	801 (60.2)	
Yes	15 (1.1)	7 (46.7)	8 (53.3)	
Alcohol drinking				**0.041**
No	1247 (92.7)	507 (40.7)	740 (59.3)	
Yes	98 (7.3)	29 (29.6)	69 (70.4)	
Having breakfast 7 days in a week				**< 0.001**
No	672 (50.0)	198 (29.5)	474 (70.5)	
Yes	673 (50.0)	338 (50.2)	335 (49.8)	
Sufficient vegetable intake				0.190
No	1156 (85.9)	452 (39.1)	704 (60.9)	
Yes	189 (14.1)	84 (44.4)	105 (55.6)	
Sufficient fruit intake				**0.001**
No	1123 (83.5)	425 (37.8)	698 (62.2)	
Yes	222 (16.5)	111 (50.0)	111 (50.0)	
Sufficient physical activity				0.490
No	1171 (87.1)	462 (39.5)	709 (60.5)	
Yes	174 (12.9)	74 (42.5)	100 (57.5)	
Excessive screen time on video				**< 0.001**
No	568 (42.2)	280 (49.3)	288 (50.7)	
Yes	777 (57.8)	256 (33.0)	521 (67.0)	
Excessive screen time on electronic game				**< 0.001**
No	659 (49.0)	309 (46.9)	350 (53.1)	
Yes	686 (51.0)	227 (33.1)	459 (66.9)	
Excessive screen time on social media				**< 0.001**
No	664 (49.4)	336 (50.6)	328 (49.4)	
Yes	681 (50.6)	200 (29.4)	481 (70.6)	
Self‐reported obesity				**0.002**
No	895 (66.5)	383 (42.8)	512 (57.2)	
Yes	450 (33.5)	153 (34.0)	297 (66.0)	
Mental toughness level				**< 0.001**
Low	723 (53.8)	256 (35.4)	467 (64.6)	
High	622 (46.2)	280 (45.0)	342 (55.0)	
Health literacy level				0.725
Low	932 (69.3)	368 (39.5)	564 (60.5)	
High	413 (30.7)	168 (40.7)	245 (59.3)	

*Note*: Bedtime late: bedtime later than or equal to 11 p.m.; Bedtime early: bedtime earlier than 11 p.m.; Sufficient vegetable intake: had ≥1.5 bowls of vegetables every day; Sufficient fruit intake: ≥2 serves of fruit every day; Sufficient physical activity: moderate/vigorous exercise ≥1 h every day; Sufficient sleep: sleep at least 8 h; Excessive screen time: spending over 2 h of screen; Mental toughness level: the mental toughness scale for adolescent (18–72) is composed of six dimensions, including challenge (3–12), commitment (3–12), emotion control (3–12), life control (3–12), confidence in abilities (3–12), and interpersonal confidence (3–12), with a higher score indicating higher mental toughness; Health literacy level: scores for evaluating personal competencies for the access to, understanding of, appraisal of, and application of health information in order to make sound decisions in everyday life; Statistical significance: p < 0.05.

Abbreviation: SES, socioeconomic status.

### 3.2 Sleeping Habits and Chi‐Square Test Results

Sixty percent of respondents (*n* = 809) reported a habitual bedtime of 11:00 p.m. or later. Late bedtime was significantly more frequent among pupils older than 14 years (*p* < 0.001), females (*p* = 0.009), those who consume alcohol (*p* = 0.041), individuals with no daily breakfast habit (*p* < 0.001), those with insufficient fruit intake (*p* = 0.001), those with excessive screen time on video/game/social media (*p* < 0.001), those who perceive themselves as obese (*p* = 0.002), and those with a lower level of mental toughness (*p* < 0.001) (Table 1). Late wake‑up (≥07:00 a.m.) was reported by 34.1% (*n* = 459). It was more common in adolescents older than 14 years (*p* = 0.002), those without a daily breakfast habit (*p* = 0.001), and those with excessive gaming time (*p* = 0.002) (Table 2).

**TABLE 2:** Participant characteristics by wake‐up time.

	Overall	Wake‐up time = Early	Wake‐up time = Late	*p*
*n*	1345	886 (65.9%)	459 (34.1%)	
Age group				**0.002**
<13 years	585 (43.5)	415 (70.9%)	170 (29.1%)	
13–14 years	379 (28.2)	241 (63.6%)	138 (36.4%)	
>14 years	381 (28.3)	230 (60.4%)	151 (39.6%)	
Sex				< 0.001
Female	757 (56.3)	541 (71.5%)	216 (28.5%)	
Male	588 (43.7)	345 (58.7%)	243 (41.3%)	
SES (0–9)				0.413
Low (0–2)	224 (16.6)	149 (66.5%)	75 (33.5%)	
Mid (3–5)	662 (49.2)	425 (64.2%)	237 (35.8%)	
High (6–9)	459 (34.1)	312 (68.0%)	147 (32.0%)	
Smoking				0.449
No	1330 (98.9)	878 (66.0%)	452 (34.0%)	
Yes	15 (1.1)	8 (53.3%)	7 (46.7%)	
Alcohol drinking				0.383
No	1247 (92.7)	817 (65.5%)	430 (34.5%)	
Yes	98 (7.3)	69 (70.4%)	29 (29.6%)	
Having breakfast 7 days in a week				**0.001**
No	672 (49.9)	414 (61.6%)	258 (38.4%)	
Yes	673 (50.1)	472 (70.1%)	201 (29.9%)	
Sufficient vegetable intake				0.620
No	1156 (86.0)	758 (65.6%)	398 (34.4%)	
Yes	189 (14.0)	128 (67.7%)	61 (32.3%)	
Sufficient fruit intake				0.058
No	1123 (83.4)	727 (64.7%)	396 (35.3%)	
Yes	222 (16.6)	159 (71.6%)	63 (28.4%)	
Sufficient physical activity				0.716
No	1171 (87.0)	774 (66.1%)	397 (33.9%)	
Yes	174 (13.0)	112 (64.4%)	62 (35.6%)	
Excessive screen time on video				0.332
No	568 (42.2)	383 (67.4%)	185 (32.6%)	
Yes	777 (57.8)	503 (64.7%)	274 (35.3%)	
Excessive screen time on electronic game				**0.002**
No	659 (49.0)	461 (69.9%)	198 (30.1%)	
Yes	686 (51.0)	425 (61.9%)	261 (38.1%)	
Excessive screen time on social media				0.721
No	664 (49.4)	441 (66.4%)	223 (33.6%)	
Yes	681 (50.6)	445 (65.3%)	236 (34.7%)	
Self‐reported obesity				0.183
No	895 (66.5)	601 (67.2%)	294 (32.8%)	
Yes	450 (33.5)	285 (63.3%)	165 (36.7%)	
Mental toughness level				>0.999
Low	723 (53.7)	476 (65.8%)	247 (34.2%)	
High	622 (46.3)	410 (65.9%)	212 (34.1%)	
Health literacy level				0.054
Low	932 (69.3)	598 (64.2%)	334 (35.8%)	
High	413 (30.7)	288 (69.7%)	125 (31.3%)	

*Note*: Wake‐up time late: wake‐up time later than or equal to 7 a.m.; Wake‐up time early: wake‐up time earlier than to 7 a.m.; Sufficient vegetable intake: had ≥1.5 bowls of vegetables every day; Sufficient fruit intake: had ≥2 serves of fruit every day; Sufficient physical activity: moderate/vigorous exercise ≥1 h every day; Sufficient sleep: sleep at least 8 h; Excessive screen time: Spending over 2 h of screen; Mental to6ughness level: the mental toughness scale for adolescent (18–72) is composed of six dimensions, including challenge (3–12), commitment (3–12), emotion control (3–12), life control (3–12), confidence in abilities (3–12), and interpersonal confidence (3–12), with a higher score indicating higher mental toughness; Health literacy level: Scores for evaluating personal competencies for the access to, understanding of, appraisal of, and application of health information in order to make sound decisions in everyday life; Statistical significance: p<0.05.

Abbreviation: SES, socioeconomic status.

Over half of the participants (56.6%; *n* = 761) obtained fewer than 8 h of sleep per night. Insufficient sleep duration was particularly prevalent among adolescents aged older than 14 years (*p* < 0.001), females (*p* < 0.001), those who consume alcohol (*p* = 0.001), individuals without a daily breakfast habit (*p* < 0.001), those with insufficient physical activity (*p* = 0.022), those with excessive screen time on video/electronic games/social media (*p* < 0.05), those who self‐reported as obese (*p* = 0.008), and those with a low level of mental toughness (*p* = 0.001) (Table 3).

**TABLE 3:** Participant characteristics by sleep duration.

	Overall	Sleep duration = Sufficient	Sleep duration = Insufficient	*p*
*n*	1345	584 (43.6%)	761 (56.6%)	
Age group				**<0.001**
<13 years	585 (43.5%)	287 (49.1%)	298 (50.9%)	
13–14 years	379 (28.2%)	172 (45.4%)	207 (54.6%)	
>14 years	381 (28.3%)	125 (32.8%)	256 (67.2%)	
Sex				**< 0.001**
Female	757 (56.3%)	288 (38.1%)	469 (61.9%)	
Male	588 (43.7%)	296 (50.3%)	292 (49.7%)	
SES (0–9)				0.562
Low (0–2)	224 (16.7%)	102 (45.5%)	122 (54.5%)	
Mid (3–5)	662 (49.2%)	278 (42.0%)	384 (58.0%)	
High (6–9)	459 (34.1%)	204 (44.4%)	255 (55.6%)	
Smoking				>0.999
No	1330 (98.9%)	577 (43.4%)	753 (56.6%)	
Yes	15 (1.1%)	7 (46.7%)	8 (53.3%)	
Alcohol drinking				**0.001**
No	1247 (92.7%)	558 (44.7%)	689 (55.3%)	
Yes	98 (7.3%)	26 (26.5%)	72 (73.5%)	
Having breakfast 7 days in a week				**< 0.001**
No	672 (50.0%)	231 (34.4%)	352 (65.6%)	
Yes	673 (50.0%)	353 (52.5%)	320 (47.5%)	
Sufficient vegetable intake				0.390
No	1156 (85.9%)	496 (42.9%)	660 (57.1%)	
Yes	189 (14.1%)	88 (46.6%)	101 (53.4%)	
Sufficient fruit intake				0.177
No	1123 (83.5%)	478 (42.6%)	645 (57.4%)	
Yes	222 (16.5%)	106 (47.7%)	116 (52.3%)	
Sufficient physical activity				**0.022**
No	1171 (87.1%)	494 (42.2%)	677 (57.8%)	
Yes	174 (12.9%)	90 (51.7%)	84 (48.3%)	
Excessive screen time on video				**< 0.001**
No	568 (42.2%)	286 (50.4%)	282 (49.6%)	
Yes	777 (57.8%)	298 (38.4%)	479 (61.6%)	
Excessive screen time on electronic games				**0.007**
No	659 (49.0%)	311 (47.2%)	348 (52.8%)	
Yes	686 (51.0%)	273 (39.8%)	413 (60.2%)	
Excessive screen time on social media				**< 0.001**
No	664 (49.4%)	357 (53.8%)	307 (46.2%)	
Yes	681 (50.6%)	227 (33.3%)	454 (66.7%)	
Self‐reported obesity				
No				**0.008**
Yes	895 (66.5%)	412 (46.0%)	483 (54.0%)	
	450 (33.5%)	172 (38.2%)	278 (61.8%)	
Mental toughness level				**0.001**
Low	723 (53.8%)	284 (39.3%)	439 (60.7%)	
High	622 (46.2%)	300 (48.2%)	322 (51.8%)	
Health literacy level				>0.828
Low	932 (69.3)	407 (43.7%)	525 (56.3%)	
High	413 (30.7)	177 (42.9%)	236 (57.1%)	

*Note*: Insufficient sleep duration: sleep duration less than 8 h; Sufficient sleep duration: sleep duration longer than or equal to 8 h; Sufficient vegetable intake: had ≥1.5 bowls of vegetables every day; Sufficient fruit intake: had ≥2 serves of fruit every day; Sufficient physical activity: moderate/vigorous exercise ≥1 h every day; Sufficient sleep: sleep at least 8 h; Excessive screen time: spending over 2 h of screen; Mental toughness level: the mental toughness scale for adolescent (18–72) is composed of six dimensions, including challenge (3–12), commitment (3–12), emotion control (3–12), life control (3–12), confidence in abilities (3–12), and interpersonal confidence (3–12), with a higher score indicating higher mental toughness; Health literacy level: scores for evaluating personal competencies for the access to, understanding of, appraisal of, and application of health information in order to make sound decisions in everyday life; Statistical significance: p < 0.05.

Abbreviation: SES, socioeconomic status.

### 3.3 Associations Between Factors and Adolescent Sleep Habits

Tables 4, 5, 6 summarize the univariable and multivariable logistic regression results examining associations between various factors and adolescent sleep habits.

**TABLE 4:** Factors associated with late bedtime among the participants.

	Crude odd ratio (95% CI)*	*p*	Adjusted odd ratio (95% CI)	*p*
Age group				
< 13	Reference		Reference	
13–14	1.36 (1.05–1.76)	0.022	1.23 (0.93–1.63)	0.153
>14	2.86 (2.16–3.80)	< 0.001	2.69 (1.98–3.64)	**< 0.001**
Sex				
Female	Reference		Reference	
Male	0.74 (0.60–0.93)	0.008	0.78 (0.61–1.01)	0.056
SES				
Low	Reference		Reference	
Medium	0.98 (0.71–1.33)	0.879	0.99 (0.70–1.38)	0.933
High	0.82 (0.59–1.14)	0.236	0.87 (0.61–1.25)	0.459
Smoking				
No	Reference		Reference	
Yes	0.76 (0.27–2.09)	0.589	0.46 (0.15–1.39)	0.169
Alcohol drinking				
No	Reference		Reference	
Yes	1.63 (1.04–2.55)	0.033	1.06 (0.65–1.73)	0.826
Having breakfast 7 days in a week				
No	Reference		Reference	
Yes	0.41 (0.33–0.52)	< 0.001	0.49 (0.39–0.63)	**< 0.001**
Sufficient vegetable intake				
No	Reference		Reference	
Yes	0.80 (0.59–1.10)	0.165	0.78 (0.55–1.11)	0.167
Sufficient fruit intake				
No	Reference		Reference	
Yes	0.61 (0.46–0.81)	0.001	0.77 (0.56–1.07)	0.118
Sufficient physical activity				
No	Reference		Reference	
Yes	0.88 (0.64–1.22)	0.440	1.11 (0.77–1.60)	0.561
Excessive screen time on video				
No	Reference		Reference	
Yes	1.98 (1.58–2.47)	< 0.001	1.46 (1.14–1.88)	**0.003**
Excessive screen time on electronic game				
No	Reference		Reference	
Yes	1.79 (1.43–2.23)	< 0.001	1.42 (1.10–1.84)	**0.007**
Excessive screen time on social media				
No	Reference		Reference	
Yes	2.46 (1.97–3.08)	< 0.001	1.78 (1.38–2.29)	**< 0.001**
Self‐reported obesity				
No	Reference		Reference	
Yes	1.45 (1.15–1.84)	0.002	1.29 (1.00–1.66)	0.051
Mental toughness level				
Low	Reference		Reference	
High	0.67 (0.54–0.83)	< 0.001	0.86 (0.67–1.11)	0.247
Health literacy level				
Low	Reference		Reference	
High	0.95 (0.75–1.21)	0.680	1.17 (0.89–1.54)	0.252

*Note*: Bedtime late: bedtime later than or equal to 23 p.m.; Bedtime early: bedtime earlier than 23 p.m.; Sufficient vegetable intake: had ≥1.5 bowls of vegetables every day; Sufficient fruit intake: had ≥2 serves of fruit every day; Sufficient physical activity: moderate/vigorous exercise ≥1 h every day; Sufficient sleep: sleep at least 8 h; Excessive screen time: spending over 2 h of screen; Mental toughness level: the mental toughness scale for adolescent (18–72) is composed of six dimensions, including challenge (3–12), commitment (3–12), emotion control (3–12), life control (3–12), confidence in abilities (3–12), and interpersonal confidence (3–12), with a higher score indicating higher mental toughness; Health literacy level: scores for evaluating personal competencies for the access to, understanding of, appraisal of, and application of health information in order to make sound decisions in everyday life; Statistical significance: p < 0.05; *: Univariable logistic regression model. Hosmer and Lemeshow goodness of fit (GOF) test: *χ* ^2^ = 7.1272, *df* = 8, *p* = 0.523.

Abbreviations: CI, confidence interval; SES, socioeconomic status.

**TABLE 5:** Factors associated with late wake‐up time among the participants.

	Crude odd ratio (95% CI)*	*p*	Adjusted odd ratio (95% CI)	*p*
Age group				
< 13	Reference		Reference	
13–14	1.40 (1.06–1.84)	0.017	1.31 (0.98–1.74)	0.065
>14	1.60 (1.22–2.10)	0.001	1.64 (1.23–2.18)	**0.001**
Sex				
Female	Reference		Reference	
Male	1.76 (1.41–2.22)	< 0.001	1.75 (1.37–2.24)	**< 0.001**
SES				
Low	Reference		Reference	
Medium	1.11 (0.81–1.53)	0.530	1.22 (0.87–1.69)	0.243
High	0.94 (0.67–1.32)	0.703	1.12 (0.79–1.60)	0.516
Smoking				
No	Reference		Reference	
Yes	1.70 (0.61–4.72)	0.308	1.42 (0.49–4.12)	0.517
Alcohol drinking				
No	Reference		Reference	
Yes	0.80 (0.51–1.25)	0.326	0.60 (0.37–0.96)	**0.032**
Having breakfast 7 days in a week				
No	Reference		Reference	
Yes	0.68 (0.55–0.86)	0.001	0.69 (0.54–0.88)	**0.002**
Sufficient vegetable intake				
No	Reference		Reference	
Yes	0.91 (0.65–1.26)	0.563	1.01 (0.72–1.43)	0.944
Sufficient fruit intake				
No	Reference		Reference	
Yes	0.73 (0.53–1.00)	0.049	0.83 (0.60–1.16)	0.285
Sufficient physical activity				
No	Reference		Reference	
Yes	1.08 (0.77–1.51)	0.654	1.07 (0.75–1.53)	0.714
Excessive screen time on video				
No	Reference		Reference	
Yes	1.13 (0.90–1.42)	0.304	1.06 (0.82–1.36)	0.664
Excessive screen time on electronic game				
No	Reference		Reference	
Yes	1.43 (1.14–1.79)	0.002	1.30 (1.01–1.67)	**0.041**
Excessive screen time on social media				
No	Reference		Reference	
Yes	1.05 (0.84–1.31)	0.679	0.96 (0.74–1.23)	0.738
Self‐reported obesity				
No	Reference		Reference	
Yes	1.18 (0.93–1.50)	0.164	1.15 (0.90–1.48)	0.254
Mental toughness level				
Low	Reference		Reference	
High	1.00 (0.80–1.25)	0.976	1.09 (0.85–1.40)	0.503
Health literacy level				
Low	Reference		Reference	
High	0.78 (0.61–1.00)	0.047	0.79 (0.60–1.04)	0.09

*Note*: Wake‐up time late: wake‐up time later than or equal to 7 a.m.; Wake‐up time early: wake‐up earlier than 7 a.m.; Sufficient vegetable intake: had ≥1.5 bowls of vegetables every day; Sufficient fruit intake: had ≥2 serves of fruit every day; Sufficient physical activity: moderate/vigorous exercise ≥1 h every day; Sufficient sleep: sleep at least 8 h; Excessive screen time: spending over 2 h of screen; Mental toughness level: the mental toughness scale for adolescent (18–72) is composed of six dimensions, including challenge (3–12), commitment (3–12), emotion control (3–12), life control (3–12), confidence in abilities (3–12), and interpersonal confidence (3–12), with a higher score indicating higher mental toughness; Health literacy level: scores for evaluating personal competencies for the access to, understanding of, appraisal of, and application of health information in order to make sound decisions in everyday life; Statistical significance: p < 0.05; *: Univariable logistic regression model. Hosmer and Lemeshow goodness of fit (GOF) test: *χ* ^2^ = 2.9258, *df* = 8, *p* = 0.9389.

Abbreviations: CI, confidence interval; SES, socioeconomic status.

**TABLE 6:** Factors associated with insufficient sleep duration among the participants.

	Crude odd ratio (95% CI)*	*p*	Adjusted odd ratio (95% CI)	*p*
Age group				
< 13	Reference		Reference	
13–14	1.16 (0.89–1.50)	0.264	1.06 (0.80–1.39)	0.696
>14	1.97 (1.51–2.58)	< 0.001	1.74 (1.31–2.31)	**< 0.001**
Sex				
Female	Reference		Reference	
Male	0.61 (0.49–0.75)	< 0.001	0.69 (0.54–0.87)	**0.002**
SES				
Low	Reference		Reference	
Medium	1.16 (0.85–1.57)	0.355	1.12 (0.81–1.55)	0.491
High	1.05 (0.76–1.44)	0.788	1.03 (0.73–1.45)	0.879
Smoking				
No	Reference		Reference	
Yes	0.88 (0.32–2.43)	0.799	0.60 (0.20–1.79)	0.362
Alcohol drinking				
No	Reference		Reference	
Yes	2.24 (1.41–3.56)	0.001	1.80 (1.10–2.96)	**0.020**
Having breakfast 7 days in a week				
No	Reference		Reference	
Yes	0.48 (0.38–0.59)	< 0.001	0.56 (0.44–0.71)	**< 0.001**
Sufficient vegetable intake				
No	Reference		Reference	
Yes	0.86 (0.63–1.17)	0.348	0.79 (0.57–1.11)	0.177
Sufficient fruit intake				
No	Reference		Reference	
Yes	0.81 (0.61–1.08)	0.155	1.00 (0.73–1.38)	0.992
Sufficient physical activity				
No	Reference		Reference	
Yes	0.68 (0.50–0.94)	0.018	0.76 (0.54–1.08)	0.129
Excessive screen time on video				
No	Reference		Reference	
Yes	1.63 (1.31–2.03)	< 0.001	1.25 (0.98–1.60)	0.078
Excessive screen time on electronic game				
No	Reference		Reference	
Yes	1.35 (1.09–1.68)	0.006	1.08 (0.84–1.38)	0.559
Excessive screen time on social media				
No	Reference		Reference	
Yes	2.33 (1.87–2.90)	< 0.001	1.87 (1.46–2.39)	**< 0.001**
Self‐reported obesity				
No	Reference		Reference	
Yes	1.38 (1.09–1.74)	0.006	1.23 (0.96–1.57)	0.096
Mental toughness level				
Low	Reference		Reference	
High	0.69 (0.56–0.86)	0.001	0.88 (0.68–1.12)	0.291
Health literacy level				
Low	Reference		Reference	
High	1.03 (0.82–1.31)	0.782	1.24 (0.95–1.62)	0.108

*Note*: Insufficient sleep duration: sleep duration less than 8 h; Sufficient sleep duration: sleep duration longer than or equal to 8 h; Sufficient vegetable intake: had ≥1.5 bowls of vegetables every day; Sufficient fruit intake: had ≥2 serves of fruit every day; Sufficient physical activity: moderate/vigorous exercise ≥1 h every day; Sufficient sleep: sleep at least 8 h; Excessive screen time: spending over 2 h of screen; Mental toughness level: the mental toughness scale for adolescent (18–72) is composed of six dimensions, including challenge (3–12), commitment (3–12), emotion control (3–12), life control (3–12), confidence in abilities (3–12), and interpersonal confidence (3–12), with a higher score indicating higher mental toughness; Health literacy level: scores for evaluating personal competencies for the access to, understanding of, appraisal of, and application of health information in order to make sound decisions in everyday life; Statistical significance: p < 0.05; *: Univariable logistic regression model. Hosmer and Lemeshow goodness of fit (GOF) test: *χ* ^2^ = 8.1726, *df* = 8, *p* = 0.4168.

Abbreviations: CI, confidence interval; SES, socioeconomic status.

As shown in Table 4, late bedtime (≥11:00 p.m.) was significantly associated with older age, self‐perceived overweight, and excessive screen time. Adolescents aged over 14 were more than twice as likely to go to bed late compared to those under 13 (aOR = 2.69, 95% CI: 1.98–3.64, *p* < 0.001). Those with exceeding screen time recommendations for video viewing (aOR = 1.46, 95% CI: 1.14–1.88, *p* = 0.003), electronic games (aOR = 1.42, 95% CI: 1.10–1.84, *p* = 0.007), and social media (aOR = 1.78, 95% CI: 1.38–2.29, *p* < 0.001) had significantly higher odds of late bedtime. In contrast, daily breakfast consumption showed a protective effect, with a 51% reduction in the odds of staying up late (aOR = 0.49, 95% CI: 0.39–0.63, *p* < 0.001).

Late wake‐up time (≥07:00 a.m.) was also more common among adolescents older than 14 years (aOR = 1.64, 95% CI: 1.23–2.18, *p* = 0.001), males (aOR = 1.75, 95% CI: 1.37–2.24, *p* < 0.001), and those with excessive gaming time (aOR = 1.30, 95% CI: 1.01–1.67, *p* = 0.041). Conversely, a daily breakfast habit (aOR = 0.69, 95% CI: 0.54–0.88, *p* = 0.002) and alcohol consumption (aOR = 0.60, 95% CI: 0.37–0.96, *p* = 0.032) were significantly associated with a lower likelihood of waking up late (Table 5).

Insufficient sleep (<8 h) was independently associated with alcohol use (aOR = 1.80, 95% CI: 1.10–2.96, *p* = 0.020), excessive social media usage (aOR = 1.87, 95% CI: 1.46–2.39, *p* < 0.001), and age older than 14 years (aOR = 1.74, 95% CI: 1.31–2.31, *p* < 0.001). On the other hand, males (aOR = 0.69, 95% CI: 0.54–0.87, *p* = 0.002) and those with a daily breakfast habit (aOR = 0.56, 95% CI: 0.44–0.71, *p* < 0.001) were significantly less likely to report inadequate sleep duration as compared to their counterparts (Table 6).

### 3.4 Sensitivity Analysis of the Association Between Mental Toughness and Sleep Habits

The findings concerning the association between the subdomains of mental toughness and sleep habits are summarized in Table S2.

Participants exhibiting higher levels of emotion control (aOR = 0.75, 95% CI: 0.59–0.96, *p* = 0.023) and commitment (aOR = 0.76, 95% CI: 0.59–0.97, *p* = 0.026) were less likely to have a late bedtime, and those with higher confidence in their abilities to confront challenges were 26% less likely to experience insufficient sleep duration (aOR = 0.74, 95% CI: 0.58–0.95, *p* = 0.017).

## 4 Discussion

### 4.1 Major Findings

This study investigated sleep habits among 1345 adolescents and identified several key findings. First, overall sleep health among adolescents was suboptimal, with 60.1% reporting late bedtimes and 56.6% reporting insufficient sleep duration, while 34.1% reported waking up late. Second, older age was consistently associated with adverse sleep outcomes, including later bedtimes, shorter sleep duration, and delayed waking. In contrast, adolescents who consumed breakfast daily were significantly less likely to exhibit these sleep problems, suggesting a potential protective role of regular dietary habits in supporting healthier sleep patterns. Third, excessive screen time emerged as a major risk factor for poor sleep, though the effects varied by screen activity type. Excessive social media use was particularly associated with sleep deprivation; extended gaming was linked to delayed wake‐up times; and all types of screen overuse were associated with later bedtimes.

### 4.2 Evidence From Prior Studies

Our study reported a concerning prevalence of poor sleeping habits among Hong Kong adolescents, including a 60.1% rate of staying up late, a 56.6% rate of insufficient sleep, and a 34.1% rate of late waking up. The adolescent sleep problem in Hong Kong has already been reported by a previous study and was found to be worsening (Chen et al. 2024). Students had a decreased average time in bed from 8.38 h in 2011–2012 to 8.08 h in 2017–2019 (Chen et al. 2024). Additionally, students experienced a 0.28‐h delay in weekday bedtime and a 0.54‐h advance in weekend wake‐up time, contributing to an overall rising trend of sleep loss (Chen et al. 2024). Adolescent sleep problems are prevalent in East Asia, with students averaging 7.1 h of weekday sleep in mainland China (Wang et al. 2021), 6.4 h in Korea (Choi et al. 2021), and 7.0 h in Japan (Kawabe et al. 2019). Adolescents in Western countries had comparatively better sleep patterns. According to a multinational study of 165,793 adolescents from 24 European and North American Countries, the average weekday sleep duration ranged from 7.47 h in Poland to 9.07 h in Flemish Belgium (Gariepy et al. 2020).

To comprehend the prevalent adolescent sleep problems in Hong Kong, this study examined several significantly associated factors. Notably, the findings indicate that older age is positively correlated with three adverse sleep habits: late bedtime, insufficient sleep duration, and late wake‐up times. A population‐based cohort study conducted in Germany revealed that increasing age was significantly related to later bedtimes and a decrease in sleep duration among children aged 4–9 years old (Lewien et al. 2021). In contrast to our findings, the German study identified a negative correlation between age and wake‐up time, possibly due to their focus on a child sample rather than an adolescent one (Lewien et al. 2021). Similarly, a Turkish questionnaire‐based study involving 3441 high school adolescents found that age was negatively associated with total night sleep duration on school nights (Yilmaz et al. 2011). Research from Saudi Arabia and the United States corroborated these results, showing a similar age‐related increase in sleep deprivation and restricted sleep (Roberts et al. 2011; Nasim et al. 2019). The age effect may be explained by older adolescents’ greater independence and autonomy over their habits, including reduced parental influence on their sleep schedules (Randler et al. 2009). Moreover, older adolescents are more likely to have access to electronic devices and engage more intensively online (Nasim et al. 2019). A US study reported that sleep duration of adolescents can be increased by advancing their bedtime, an effect that remains consistent for adolescents aged between 10 and 21 years (Campbell et al. 2023). Therefore, parents and policy‐makers should consider mitigating the adolescent sleep problem by making adolescents to go to bed early.

The study also identified that adolescents who consumed breakfast daily were less likely to exhibit poor sleep habits. This is consistent with findings from the DELICIOUS project, which demonstrated a positive association between breakfast consumption and adequate sleep duration (Godos et al. 2025). Additionally, other dietary factors, such as eating at school and maintaining a higher diet quality, were identified as being linked to sufficient sleep duration (Godos et al. 2025). The relationship between breakfast consumption and sleep quality may be influenced by glucose instability caused by skipped meals, which can disrupt both sleep initiation and maintenance (Kobayashi et al. 2014). However, the impact of breakfast consumption on adolescent sleep quality remained controversial. A US intervention study found that although breakfast consumption reduced evening intake of high‐carbohydrate, high‐fat, and sugary foods, it paradoxically resulted in shorter total sleep time compared to skipping breakfast (Gwin and Leidy 2018). Nonetheless, the reduction in total sleep duration was not considered detrimental, as sleep efficiency remained unaffected, and both perceived sleep quality and sleep onset latency improved (Gwin and Leidy 2018). On the other hand, St‐Onge et al. reported that behaviors linked to breakfast skipping, such as increased energy intake from sugar and carbohydrates, were associated with poor sleep quality (St‐Onge et al. 2016).

This study also highlighted the detrimental effects of excessive screen activity on sleep habits, with varying impacts depending on the type of screen activity. Specifically, excessive social media screen time was associated with an increased risk of sleep deprivation, while excessive gaming was linked to a higher likelihood of waking up late. Furthermore, increased screen time across all types was positively correlated with late bedtimes. Similarly, a cross‐sectional study from Japan found that students addicted to the internet had significantly shorter sleep durations and delayed bedtimes compared to those with less screen time (Kawabe et al. 2019). A Swedish study of 278 students identified nighttime messaging as a major factor contributing to delayed bedtimes, sleep deprivation, daytime tiredness, and disrupted sleep schedules (Garmy and Ward 2018). Regarding the electronic game screen time, many studies have found it to be negatively associated with sleep quality (King et al. 2013; Weaver et al. 2010). An Australian study found that excessive screen time on violent video games was associated with decreased sleep efficiency and total sleep time, even when students adhered to normal bedtimes after gaming (Yilmaz et al. 2011). Another study suggested that the effect of gaming on sleep quality was more closely linked to playing intensity than to duration (Altintas et al. 2019). A 2022 systematic review concluded that the use of electronic devices among adolescents was associated with sleep problems and could lead to mental health issues (de Sá et al. 2023). Additionally, poor sleep quality combined with video game engagement was significantly associated with abdominal adiposity (Turel et al. 2017). Consequently, it is important for both parents and policy‐makers to regulate screen time among adolescents to protect their health and well‐being. Although this study found no significant association between mental toughness levels and detrimental sleep patterns, further analysis indicated that certain attributes of adolescent mental toughness—such as confidence in abilities during challenging circumstances, emotional regulation, and a strong belief in commitment—serve as protective factors against such adverse sleep behaviors. The relationship between these attributes and stress has been corroborated in prior research (Alam and Mohanty 2023; McCann and Silvers 2025; Gunes and Yetim 2023), with existing evidence suggesting that individuals are more likely to experience adverse sleep habits when experiencing stress, including delayed bedtimes and reduced sleep duration (Schmidt et al. 2024). Consequently, this phenomenon may be explained by the mediating role of mental toughness attributes in the connection between stress and unhealthy sleep practices.

Gender differences in sleep patterns were also evident in our study, with females more likely to experience insufficient sleep duration and males more likely to exhibit delayed wake‐up times. This aligns with a national study in New Zealand, which reported higher prevalence of poor sleep quality and worse sleep hygiene among girls (Galland et al. 2017). An Australian study also found that poor sleep quality was more prevalent in females compared to males (Fatima et al. 2016). The impact of short sleep duration and poor sleep quality on academic and social activities is believed to be more detrimental for girls (Forest et al. 2022). Furthermore, a US survey revealed no significant gender differences in total sleep time, although girls were found to wake up earlier than boys (Lee et al. 1999). Our study reported that alcohol consumption was associated with less likelihood of waking up late and increased possibility of insufficient sleep duration. Many previous studies have found early use of alcohol among adolescents to be associated with shorter sleep duration and poorer sleep quality (Mike et al. 2016; Marmorstein 2017; Zheng et al. 2021).

### 4.3 Strengths and Limitations

Regarding strengths of this study, first, it is based on a relatively large sample of adolescents, allowing for adequate statistical power to detect associations between multiple behavioral and psychosocial factors and sleep outcomes. Second, the study employed a comprehensive set of validated and theory‐informed instruments to assess key variables. Third, sleep was examined multidimensionally, encompassing bedtime, wake‐up time, and sleep duration, which offers a more nuanced understanding of adolescent sleep behavior. Finally, the use of multivariable logistic regression models allowed for adjustment of important covariates, strengthening the robustness of the findings.

However, several limitations should be acknowledged. The cross‐sectional design precludes causal inferences, and reverse causality between sleep patterns and behavioral factors (e.g., screen time or dietary habits) cannot be ruled out. Although associations between the primary outcome and covariates were observed in this study, previous research has emphasized the influence of potential interrelationships and reverse causation. For example, an association between healthy eating habits and sound sleep habits was identified, whereas one study reported a positive association between sleep interventions and eating behavior habits (Fenton et al. 2021). Likewise, the interrelationship between physical activity and screen time has been observed (Aulbach et al. 2023; Lizandra et al. 2019). This phenomenon reveals complex interactions among variables and the impact of confounders. Comprehending this reverse causation is essential for elucidating the actual effect of health behaviors. Notably, despite the potential for reverse causation, these findings further underscore the significance of interventions aimed at sleep duration, as such measures may contribute to the enhancement of multiple health behavior indicators. All data were self‐reported, which may be subject to recall and social desirability biases, particularly for sensitive behaviors such as alcohol use or perceived weight status. The sleep outcomes were assessed via self‐reported typical patterns rather than objective measures (e.g., actigraphy), potentially limiting measurement precision. However, in this study, all participants were explicitly informed of the confidentiality of the study and that the questionnaires would not be available for review by teacher or schools, in order to minimize potential bias. In addition, the study sample was recruited based on purposive sampling, which may limit the generalizability of our findings to broader adolescent populations. Potential selection bias may exist, as schools and students who consent to join might be systematically different from those who did not participate. Lastly, while a wide range of covariates was included, unmeasured confounders such as parental sleep behavior, mental health conditions, or environmental noise could have influenced sleep outcomes.

### 4.4 Implications

Our study revealed the unsatisfactory sleeping habits among Hong Kong adolescents, underscoring the urgent need for public health interventions. For example, since there are strong associations between screen time—particularly social media use—and adverse sleep outcomes, policy‐makers should consider introducing or strengthening age‐appropriate screen time guidelines in schools or at the community. Public health campaigns targeting digital hygiene, including time‐restricted screen use and digital curfews, may be beneficial. In parallel, integrating sleep education into existing school‐based health curricula may raise awareness of the importance of adequate and consistent sleep. For future research, longitudinal studies are needed to assess the temporal relationships and potential causal pathways between lifestyle factors and sleep outcomes in adolescents. Additionally, the use of objective sleep measures (e.g., actigraphy) and the inclusion of environmental, familial, and psychological variables will enhance understanding of the multifaceted determinants of adolescent sleep health.

## 5 Conclusions

In conclusion, this study found that 60.1% of adolescents reported late bedtime, 56.6% had insufficient sleep duration, and 34.1% reported waking up late, indicating a high prevalence of poor sleep habits. Multivariable analyses identified older age and excessive screen use as key risk factors, while daily breakfast consumption emerged as a consistent protective factor. These findings underscore the need for targeted interventions—such as digital hygiene promotion and school‐based sleep education—to foster healthier sleep routines among adolescents.

## Author Contributions

Claire Chenwen Zhong, Junjie Huang, and Martin C. S. Wong conceptualized and supervised the study. Zhaojun Li, Vera M. W. Keung, Amelia Lo, and Calvin Cheung were responsible for data curation and formal analysis. Claire Chenwen Zhong, Mingtao Chen, and Zehuan Yang drafted the manuscript. Junjie Huang, and Martin C. S. Wong reviewed and revised the manuscript. All authors read and approved the final manuscript.

## Ethics Statement

The Survey and Behavioural Research Ethics Committee of the Chinese University of Hong Kong approved the study (approval no. SBRE‐24‐0029).

## Conflicts of Interest

Martin C. S. Wong is an honorary medical advisor of GenieBiome Ltd., SunRise, and BGI Health. He is an advisory committee member of Pfizer; is an external expert of GlaxoSmithKline Limited; is a member of the advisory board of AstraZeneca; and has been paid consultancy fees for providing advice on research. He has received grants from Pfizer, Merck Sharp & Dohme, AstraZeneca, GSK, and Moderna. The other authors declare no conflicts of interest.

## Data Sharing Statement

The datasets used and/or analyzed during the current study are available from the corresponding author upon reasonable request.

## Peer Review

The peer review history for this article is available at https://publons.com/publon/10.1002/brb3.70885

## Supporting information




**Supplementary Material**: brb370885‐sup‐0001‐SuppMat.docx
