# Supplementary material for: Examining Sleep Habits and Associated Lifestyle Factors in Adolescents: A Population‐Based Study
Source: Brain Behav. 2025 Oct 9;15(10):e70885. doi: 10.1002/brb3.70885 (PMC12510639; doi:10.1002/brb3.70885)
Supplement: Supplementary file 1 — Supplementary Material: brb370885‐sup‐0001‐SuppMat.docx [file BRB3-15-e70885-s001.docx]

Supplementary Material

Supplementary Table 1. Survey Instrument

Supplementary Table 2. Result of the Association Between MTS-A Sub-domain and Sleep Habits Pattern

**Supplementary Table 1. Survey Instrument**

# FASII

Does your family own a car, van, or truck?

| ☐ No | ☐ Yes, one | ☐ Yes, two or more |
| --- | --- | --- |

Do you have your own bedroom?

| ☐ No | ☐ Yes |
| --- | --- |

During the past 12 months, how many times did you travel away on holiday with your family?

| ☐ Not at all | ☐ Once | ☐ Twice | ☐ More than twice |
| --- | --- | --- | --- |

How many computers does your family own?

| ☐ None | ☐ One | ☐ Two | ☐ More than two |
| --- | --- | --- | --- |

# Health Literacy

The following questions ask about your perceived abilities and skills in accessing and using health information. Please select the option that best describes each of the following abilities or skills and answer all the questions.

| **Items** | Never | Rarely | Sometimes | Usually | Always |
| --- | --- | --- | --- | --- | --- |
| I try to get more information about health as much as possible | ☐ | ☐ | ☐ | ☐ | ☐ |
| I am able to find health information that I need | ☐ | ☐ | ☐ | ☐ | ☐ |
| When ill or facing health problems, I can get the necessary information I need | ☐ | ☐ | ☐ | ☐ | ☐ |
| I am able to ask others about health information that I need | ☐ | ☐ | ☐ | ☐ | ☐ |
| I am able to access information about the healthy diet that is appropriate for my age group | ☐ | ☐ | ☐ | ☐ | ☐ |
| I am able to access information about the physical activity appropriate for my age group | ☐ | ☐ | ☐ | ☐ | ☐ |
| I am able to access information about the proper care required for my skin and hair that is appropriate for my age group | ☐ | ☐ | ☐ | ☐ | ☐ |
| I am able to access information about mental health appropriate for my age group | ☐ | ☐ | ☐ | ☐ | ☐ |
| I am able to find useful resources about health Information on the Internet | ☐ | ☐ | ☐ | ☐ | ☐ |
| I can read brochures on prescribed medicine | ☐ | ☐ | ☐ | ☐ | ☐ |
| I can easily read educational brochures about nutritional issues | ☐ | ☐ | ☐ | ☐ | ☐ |
| I can easily read brochures/fact sheets about disease prevention (e.g. anaemia, osteoporosis, respiratory infections, etc.) | ☐ | ☐ | ☐ | ☐ | ☐ |
| I can easily read health information materials in magazines and newspapers | ☐ | ☐ | ☐ | ☐ | ☐ |
| I can easily read health information materials on the Internet (e.g. websites) | ☐ | ☐ | ☐ | ☐ | ☐ |
| I can easily understand the meaning of the signs used in hospitals and medical centres | ☐ | ☐ | ☐ | ☐ | ☐ |
| I can understand most things I hear about health | ☐ | ☐ | ☐ | ☐ | ☐ |
| I can easily understand the content of health information that I find | ☐ | ☐ | ☐ | ☐ | ☐ |
| I can easily understand my doctor’s instructions and recommendations (e.g. prescriptions) | ☐ | ☐ | ☐ | ☐ | ☐ |
| I can easily understand information about medications – usage, side effects and warnings | ☐ | ☐ | ☐ | ☐ | ☐ |
| I can easily understand the nutrition facts on food packages | ☐ | ☐ | ☐ | ☐ | ☐ |
| I can understand the information and recommendations about proper nutrition for adolescents in the media (e.g. radio, TV, internet, etc.) | ☐ | ☐ | ☐ | ☐ | ☐ |
| I can understand the information and warnings provided by the media (e.g. radio, TV, internet, etc.) about tobacco, drug abuse and risky behaviours | ☐ | ☐ | ☐ | ☐ | ☐ |
| I can understand the information and recommendations about health and illness in the media | ☐ | ☐ | ☐ | ☐ | ☐ |
| I can understand the recommendations on prevention of accidents and injuries | ☐ | ☐ | ☐ | ☐ | ☐ |
| When faced with new health information, I can judge its accuracy | ☐ | ☐ | ☐ | ☐ | ☐ |
| I would compare the data obtained from various sources | ☐ | ☐ | ☐ | ☐ | ☐ |
| When dealing with conflicting information about health issues, I can recognize the correct information | ☐ | ☐ | ☐ | ☐ | ☐ |
| I have the ability to judge which resources I can trust | ☐ | ☐ | ☐ | ☐ | ☐ |
| When dealing with nutritional information I can choose the right information | ☐ | ☐ | ☐ | ☐ | ☐ |
| When shopping, I choose food based on its nutrition facts (e.g. amount of energy, sugar, protein, etc.) written on the packaging | ☐ | ☐ | ☐ | ☐ | ☐ |
| I try to choose foods without preservatives | ☐ | ☐ | ☐ | ☐ | ☐ |
| I try to apply what I have learned about health issues in my everyday life | ☐ | ☐ | ☐ | ☐ | ☐ |
| I try to keep my body weight in balance | ☐ | ☐ | ☐ | ☐ | ☐ |
| I can discuss my concerns relating to health issues with health providers | ☐ | ☐ | ☐ | ☐ | ☐ |
| When visiting a doctor or health provider I am able to give him/her all of my necessary personal information | ☐ | ☐ | ☐ | ☐ | ☐ |
| When visiting a doctor or health provider I am able to tell him/her the name of the medications that I have previously used | ☐ | ☐ | ☐ | ☐ | ☐ |
| When visiting a doctor or health provider I am able to ask all the questions I have | ☐ | ☐ | ☐ | ☐ | ☐ |
| I can share the health information that I gather with others (e.g. family, friends, etc.) | ☐ | ☐ | ☐ | ☐ | ☐ |
| If I have any questions about health issues I am able to get information and advice from others | ☐ | ☐ | ☐ | ☐ | ☐ |
| When visiting a doctor or health provide I am able to ask questions based on my research | ☐ | ☐ | ☐ | ☐ | ☐ |
| I talk to my friends about avoiding risky behaviour (e.g. smoking, hookah, drugs, etc.) | ☐ | ☐ | ☐ | ☐ | ☐ |

| **Nutrition facts** |
| --- |
| **Serving size:** 1 cup (240 cc) |
| **Servings per container**: 4 |
| **Amount per serving:** |
| **Energy**: 140 Kcal |
| **Total Fat:** 7gr  **Cholesterol:** 30 mg |
| **Carbohydrates:** 11gr  **Sugar:** 0 gr |
| **Protein**: 8gr |
| **Sodium:** 160 mg |

This information is on the back of a container of milk. If a person drinks 3 cups of milk in one given day, how many carbohydrates has he/she received?

Answer: ­­­­­­­­­­­­­­­­­­­­­­­­­­­­­__________________

Calculate the BMI of a person with height=160 cm and weight=70 kg?

BMI= $\frac{weight(kg)}{height{(m)}^{2}}$

Answer: ­­­­­­­­­­­­­­­­­­­­­­­­­­­­­__________________

What is this person’s body fat status (based on the following information)?

|  | **Underweight** | **Normal weight** | **Overweight** | **Obese** |
| --- | --- | --- | --- | --- |
| **BMI** | **<18.5** | **18.5-24.9** | **25-29.9** | **≥30** |

| ☐ Underweight | ☐ Normal weight |
| --- | --- |
| ☐ Overweight | ☐ Obese |

# Dietary Habit

In the past 7 days, how much vegetables did you eat on average per day?

| ☐ Never | ☐ Less than half a bowl per day | ☐ Half bowl to one bowl per day |
| --- | --- | --- |
| ☐ 1 to less than 1.5 bowls per day | ☐ 1.5 or more bowls per day |  |

In the past 7 days, how much fruit did you eat on average per day? Each serving of fruit refers to one medium-sized fruit (such as a kiwi), half a large-sized fruit (such as a dragon fruit), or half a bowl of fruit (such as grapes).

| ☐ Never | ☐ Less than half a serving per day | ☐ Half a serving to a serving per day |
| --- | --- | --- |
| ☐ 1 to less than 2 servings per day | ☐ 2 or more servings per day |  |

In the past 7 days, on how many days did you eat breakfast?

| ☐ Never | ☐1-2 days | ☐3-4 days | ☐5-6 days | ☐Everyday |
| --- | --- | --- | --- | --- |

# Physical Activity:

In the past 7 days, how much time did you spend on moderate to vigorous intensity physical activity in total?

(Moderate intensity means that breathing becomes rapid during activity, but one can still speak normally, such as cycling, brisk walking, playing table tennis, or playing in the park; Vigorous intensity means that breathing becomes rapid during activity that one cannot speak normally, such as playing soccer, jumping rope, running, or playing basketball.)

| ☐ Never | ☐ Less than 1 hour | ☐ 1 to less than 2 hours |
| --- | --- | --- |
| ☐ 2 to less than 3 hours | ☐ 3 to less than 4 hours | ☐ 4 to less than 5 hours |
| ☐ 5 to less 6 hours | ☐ 6 to less than 7 hours | ☐ At least 7 hours |

# Screen time:

In general, how much time do you spend watching TV or videos on an electronic product every day on weekdays?

| ☐ Never | ☐ Less than 1 hour per day | ☐ 1 to less than 2 hours per day |
| --- | --- | --- |
| ☐ 2 to less than 3 hours per day | ☐ 3 to less than 4 hours per day | ☐ 4 to less than 5 hours per day |
| ☐ 5 to less than 6 hours per day | ☐ 7 to less than 8 hours per day | ☐ At least 8 hours per day |

In general, how much time do you spend playing electronic games every day on weekdays?

| ☐ Never | ☐ Less than 1 hour per day | ☐ 1 to less than 2 hours per day |
| --- | --- | --- |
| ☐ 2 to less than 3 hours per day | ☐ 3 to less than 4 hours per day | ☐ 4 to less than 5 hours per day |
| ☐ 5 to less than 6 hours per day | ☐ 7 to less than 8 hours per day | ☐ At least 8 hours per day |

In general, how much time do you spend using social media every day on weekdays?

| ☐ Never | ☐ Less than 1 hour per day | ☐ 1 to less than 2 hours per day |
| --- | --- | --- |
| ☐ 2 to less than 3 hours per day | ☐ 3 to less than 4 hours per day | ☐ 4 to less than 5 hours per day |
| ☐ 5 to less than 6 hours per day | ☐ 7 to less than 8 hours per day | ☐ At least 8 hours per day |

# Sleep habits:

In the past month, what time did you usually go to bed at night?

| ☐ 9:00 p.m. | ☐ 9:30 p.m. | ☐ 10:00 p.m. |
| --- | --- | --- |
| ☐ 10:30 p.m. | ☐ 11:00 p.m. | ☐ 11:30 p.m. |
| ☐ 12:00 a.m. (Midnight). | ☐ 12:30 a.m. | ☐ 1:00 a.m, |
| ☐ 1:30 a.m. | ☐ Other:__________ |  |

In the past month, what time did you usually wake up in the morning?

| ☐ 5:00 a.m. | ☐ 5:30 a.m. | ☐ 6:00 a.m. |
| --- | --- | --- |
| ☐ 6:30 a.m. | ☐ 7:00 a.m. | ☐ 7:30 a.m. |
| ☐ 8:00 a.m. | ☐ 8:30 a.m. | ☐ Other:__________ |

# Self-rate obese:

How would you describe your weight?

| ☐ Very underweight |
| --- |
| ☐ Slightly underweight |
| ☐ About the right weight |
| ☐ Slightly overweight |
| ☐ Very overweight |

# Smoking and Alcohol Consumption

The following question is related to smoking and alcohol drinking. Your response will not be reviewed by anyone other than the research staff.

Have you ever tried smoking?

| ☐ Yes | ☐No |
| --- | --- |

During the past 30 days, on how many days did you smoke cigarettes?

| ☐ 0 days | ☐ 1-2 days | ☐ 3-5 days |
| --- | --- | --- |
| ☐ 6-9 days | ☐ 10-19 days | ☐ 20-29 days |
| ☐ All 30 days |  |  |

Have you ever tried drinking alcohol?

| ☐ Yes | ☐No |
| --- | --- |

During the past 30 days, on how many days did you drink alcohol?

| ☐ 0 days | ☐ 1-2 days | ☐ 3-5 days |
| --- | --- | --- |
| ☐ 6-9 days | ☐ 10-19 days | ☐ 20-29 days |
| ☐ All 30 days |  |  |

# Mental Toughness Scale

The following section asks for your thoughts and feelings as student. No right or Wrong answers were set, and please choose the appropriate answer to reflect your level of agreement with each statement

| Items | Strongly Disagree | Disagree | Agree | Strongly Agree |
| --- | --- | --- | --- | --- |
| I find it difficult to stop myself getting angry/upset/stressed | ☐ | ☐ | ☐ | ☐ |
| I feel confident in social situations | ☐ | ☐ | ☐ | ☐ |
| When faced with difficulties, I usually give up | ☐ | ☐ | ☐ | ☐ |
| It’s always good to try challenging things | ☐ | ☐ | ☐ | ☐ |
| I feel in control of what happens in my life | ☐ | ☐ | ☐ | ☐ |
| Challenges bring out the best in me | ☐ | ☐ | ☐ | ☐ |
| I am good at managing negative emotions (e.g. anger, sadness, worry) | ☐ | ☐ | ☐ | ☐ |
| I feel confident speaking in front of other people | ☐ | ☐ | ☐ | ☐ |
| If I work hard, my future can be whatever I want it to be | ☐ | ☐ | ☐ | ☐ |
| I cannot control what will happen in my future | ☐ | ☐ | ☐ | ☐ |
| I believe in my own abilities | ☐ | ☐ | ☐ | ☐ |
| I feel nervous around new people | ☐ | ☐ | ☐ | ☐ |
| I am happy to try new and challenging tasks | ☐ | ☐ | ☐ | ☐ |
| I give up if I’m under pressure | ☐ | ☐ | ☐ | ☐ |
| I leave many things unfinished | ☐ | ☐ | ☐ | ☐ |
| In general, I am confident in my abilities | ☐ | ☐ | ☐ | ☐ |
| My emotions (e.g. anger, sadness, worry) sometime take control of me | ☐ | ☐ | ☐ | ☐ |
| In general, I lack confidence in my ability | ☐ | ☐ | ☐ | ☐ |

| MTS-A sub-domain | Late Bedtime | |  | Late Wake up | |  | Insufficient Sleep Duration | |
| --- | --- | --- | --- | --- | --- | --- | --- | --- |
|  | aOR (95% CI) | P-value |  | aOR (95% CI) | P-value |  | aOR (95% CI) | P-value |
|  |  |  |  |  |  |  |  |  |
| Challenge |  |  |  |  |  |  |  |  |
| Low | Ref |  |  | Ref |  |  | Ref |  |
| High | 0.93 (0.69-1.25) | 0.623 |  | 1.08 (0.80-1.46) | 0.626 |  | 0.83 (0.62-1.11) | 0.204 |
| Interpersonal confidence |  |  |  |  |  |  |  |  |
| Low | Ref |  |  | Ref |  |  | Ref |  |
| High | 0.91 (0.71-1.16) | 0.452 |  | 1.14 (0.89-1.45) | 0.300 |  | 0.95 (0.74-1.20) | 0.651 |
| Confidence in abilities |  |  |  |  |  |  |  |  |
| Low | Ref |  |  | Ref |  |  | Ref |  |
| High | 0.79 (0.62-1.02) | 0.075 |  | 1.14 (0.89-1.47) | 0.302 |  | 0.74 (0.58-0.95) | **0.017** |
| Emotion control |  |  |  |  |  |  |  |  |
| Low | Ref |  |  | Ref |  |  | Ref |  |
| High | 0.75 (0.59-0.96) | **0.023** |  | 1.14 (0.90-1.46) | 0.273 |  | 0.84 (0.66-1.06) | 0.146 |
| Life control |  |  |  |  |  |  |  |  |
| Low | Ref |  |  | Ref |  |  | Ref |  |
| High | 0.80 (0.60-1.06) | 0.118 |  | 0.91 (0.69-1.22) | 0.535 |  | 1.06 (0.80-1.40) | 0.679 |
| Commitment |  |  |  |  |  |  |  |  |
| Low | Ref |  |  | Ref |  |  | Ref |  |
| High | 0.76 (0.59-0.97) | **0.026** |  | 1.14 (0.89-1.45) | 0.298 |  | 0.80 (0.63-1.02) | 0.067 |

**Supplementary Table 2. Result of the Association Between MTS-A Sub-domain and Sleep Habits Pattern**

MTS-A: Mental Toughness Scale for Adolescents; Late Bedtime: bedtime of 23:00 or later; Late Wake Up: Wake-up of 07:00 or later; Insufficient sleep duration: Fewer than 8 hours’ sleep per night; aOR: Adjusted odds ratio; CI: Confidence Intervals
